# Pharmacist and student evaluation of a preceptor training program in a regional Australian University: a multi-method study

**DOI:** 10.1186/s12909-023-04979-7

**Published:** 2024-01-10

**Authors:** Gillian J Knott, Martina F Mylrea, Beverley D Glass

**Affiliations:** Pharmacy, College of Medicine and Dentistry, James Cook University, Townsville, Queensland Australia

**Keywords:** Experiential education, Program evaluation, Preceptor training, Pharmacist preceptor

## Abstract

**Background:**

Increased emphasis on workplace-based learning within pharmacy curricula has led to a focus on the quality of preceptors and the provision of preceptor training, with a diverse range of training programs for preceptors being developed across the globe. To ensure that preceptors are trained appropriately and deemed to be competent in their role, it is essential that all training programs are suitably evaluated. This research aimed to evaluate an online preceptor training program at a regional Australian University.

**Methods:**

Kirkpatrick’s four level model for assessment of training was used to evaluate this program. A multi method approach included a preceptor post training survey and interviews and a student survey evaluating the preceptor. Preceptor survey data were analysed using descriptive statistics and content analysis, while inductive thematic analysis was used to analyse the interviews. Student evaluations of trained and untrained preceptors were compared to determine whether training had impacted on student-rated preceptor effectiveness.

**Results:**

Twenty-eight preceptor post-training surveys were received, ten preceptor post-training interviews were conducted, and 35 student surveys were completed. The program was rated positively overall, with notable mention by preceptors of the interactive networking session. Following their first post-training student placement, preceptors found that their overall confidence levels had improved, particularly in relation to student management, evaluating students and providing feedback. Student evaluations of preceptors revealed improved ratings of trained versus untrained preceptors, especially as effective communicators.

**Conclusions:**

This study demonstrated that training had a positive impact on preceptor attitudes, behaviour and confidence levels. From the perspective of the student, training was also found to improve preceptor performance. These results highlight the beneficial effects of training for preceptors, to optimize the student placement experience and their preparation for future practice.

## Background

With the current focus on producing work-ready pharmacy graduates, a significant amount of experiential placement is embedded within pharmacy curricula, highlighting the important role of pharmacist preceptors, particularly as a role model and linking theory to practice [1–3]. Preceptors, as clinical supervisors, are often challenged by their ability to combine their role as a student preceptor and the fulfilment of their professional responsibilities as a pharmacist, in the provision of high-quality service and advice to patients. Furthermore, while they may be knowledgeable and highly qualified in their field, they do not necessarily have skills in the areas of learning and teaching, assessment, and providing feedback [4, 5]. They may also have a limited understanding of the university curriculum and their roles and responsibilities as a preceptor, both of which are necessary to identify and address the specific needs of the student. Training is therefore strongly recommended for preceptors, to ensure that they are informed, confident and competent in their precepting role [6, 7].

Documented preceptor training programs reported in the literature are diverse in nature, with some programs being developed for all preceptors, and some being specific for intern, resident or student preceptors. Programs may have a focus on community or hospital pharmacy and may be designed for a single program, a single university or for a consortium of universities [8, 9]. In general, it is accepted that due to the diversity of both preceptors and placement sites, programs should be tailored to requirements of the specific program or institution, although there should be some standardization of key program elements [1–3, 8, 10]. Programs can vary widely in both structure and content and may include face-to-face or online delivery methods, with flexibility of attendance being desirable [1, 8, 10]. Program design should consider preceptor roles and responsibilities, relevant preceptor competency standards and the requirements of the program or university, including the availability of program resources. The requirements of professional and accrediting bodies and the individual needs of the preceptor should also be considered. [3] While preceptor training and development programs are now a component of most university health programs, there remains no consensus on the ideal structure and content of such programs [6, 8, 10]. Once developed, it is recommended that all preceptor training programs are appropriately evaluated to ensure that they are fit for purpose and have met the needs of both the preceptors and the students.

While a range of preceptor training programs have been documented in the literature [8, 9], there are few examples of program evaluations, with most conducted from the perspective of the preceptor only. These evaluations have included quantitative post-training surveys, qualitative post-training interviews, post-training focus groups or discussions and pre and post training surveys [11–18].

In line with Kirkpatrick’s model of training evaluation criteria, a comprehensive program evaluation should identify the effect of the program on the preceptor, in terms of their learning and changes related to their precepting practices, but also to ascertain whether these changes have improved outcomes with respect to both preceptor practice and student performance [19]. This study reports on the evaluation of a community pharmacy preceptor training program, which was implemented at James Cook University (JCU) in Australia in 2022. Community preceptors represent approximately 70% of the preceptor cohort at JCU and were the specific focus of this program, due to the increased difficulties faced by preceptors in the community pharmacy environment, with considerable demands on their time and a highly variable workload. This study aimed to evaluate the impact and outcome of the preceptor training program on:

1. Preceptor self-reported learning, confidence levels and precepting behaviours.
2. Preceptor performance through student evaluations of their preceptor.

## Methods

James Cook University requires pharmacy students to complete 600 h of experiential placement during their BPharm (Hons) degree, mainly in the third and fourth year of their degree, under the supervision of a pharmacist preceptor. In 2022, an online preceptor training program was designed, developed and implemented for JCU community pharmacy preceptors. The design of the program was informed by a comprehensive preceptor training needs analysis and collaboration with a preceptor training expert advisory group. Members of the advisory group included the research team, two JCU pharmacist preceptors, and a representative from both the Pharmaceutical Society of Australia and the Australian Pharmacy Council (APC) [20, 21]. The program consisted of four flexible learning modules based on the main preceptor roles (Role Model, Educator, Assessor, Mentor) and a small group online interactive networking session, with optional participation in an online asynchronous discussion forum. Each module contained information relating to the particular role of the preceptor, and included information, video clips, online activities and a small quiz to reflect on the knowledge gained from the module. Modules were designed to allow preceptors to progress at their own pace over a period of 5 weeks, with an estimated total completion time of six hours. The interactive networking activity allowed for small group case-based discussion and promoted the sharing of precepting experiences [22]. Sixty-two (25.8%) of the JCU cohort of community pharmacist preceptors enrolled in the training program and 28 preceptors completed the program.

Following implementation, the program was evaluated using Kirkpatrick’s model of training evaluation criteria (reaction, learning, behaviour, results) as a guide [19]. This evaluation included a preceptor post-training survey, preceptor post-training interviews and a survey comparing student evaluations of trained and untrained preceptors.

### Preceptor post-training survey

An anonymous survey was administered using the Qualtrics® survey platform to all 28 pharmacist preceptors, who had completed the training program. This survey aimed to address levels 1 and 2 of Kirkpatrick’s model, which focuses on the participant reaction to the program and the level of learning achieved through the program. Preceptors were invited to participate through an online link within the preceptor training online organizational site. They were advised of the estimated survey completion time of up to 15 min.

The survey employed a 5-point Likert-type scale to ascertain overall preceptor views on the adequacy and relevance of the program content and the appropriateness and convenience of the program format for their current needs. Preceptors were then asked to rate the usefulness of each individual component of the program, again using a 5-point Likert-type scale to grade their responses. Two open response questions asked preceptors to comment on the best aspects of the training program and any suggested improvements. Respondents were asked to provide details of their past precepting experience and training, to allow for comparisons between preceptor experience and their responses.

Survey data were transferred into SPSS® (SPSS 27 Statistics for Windows, Armonk, NY: IBM Corp) and analysis was conducted using descriptive statistics. To identify any association between preceptor responses and past precepting experience, Chi-square tests were also conducted, with significance level being set at α = 0.05. Conceptual content analysis was used to determine and quantify common themes from the open response questions. The open comments were collated, then themed into appropriate categories, with the themes being presented in order of frequency of occurrence, along with the respective preceptor quotes. The results of the content analysis were then related back to the survey results.

### Preceptor post-training post-placement interviews

Interviews were conducted with all preceptors who received a placement student within eight months of completing preceptor training. The aim of these interviews was to assess the longer-term outcomes of the training program and evaluate any self-reported changes to precepting behaviour that may have resulted from preceptor training. This will address level 3 of Kirkpatrick’s model of training evaluation criteria (behaviour). Preceptors who had completed the training program and had also hosted a student post-training, were identified through the JCU placement program records. Each preceptor was invited by email to participate in a semi-structured telephone interview, with an estimated duration of 15 to 20 min. Preceptors were asked about any challenges that they experienced when supervising their last placement student, then questioned about the effect of preceptor training on their attitudes and practices, and their confidence levels as a preceptor. Finally, preceptors were asked to identify additional topics or support measures, which could be included in future programs. Background information on their precepting history and previous training attendance was also collected, to enable comparisons to be made during data analysis.

Interviews were recorded, transcribed verbatim and then analysed according to Braun and Clarke’s method of thematic analysis [23], using NVivo® (NVivo; QSR international Pty Ltd, Version 12, 2018). Data were coded and themes were identified within these codes, which were then presented in the manuscript, along with illustrative quotes.

### Student evaluation of the preceptor

Following each experiential placement, all JCU students are required to evaluate their preceptor via a ‘*Student evaluation of the preceptor’* anonymous survey. In this survey, students are asked to rate their preceptor using a 5-point Likert scale as 1, Poor; 2, Average; 3, Good; 4, Very Good; or 5, Excellent, for 18 different precepting skills. Two demographic questions are included to ascertain the nature of their placement site (Community pharmacy, Hospital pharmacy or Other). At the end of the survey, students are invited to make open comments about their preceptor.

Student surveys were collected in the year following training. Due to the anonymity of the student survey, for the purpose of identifying preceptors who had been trained, students were additionally asked in the post-training evaluation survey to state whether their preceptor had completed the JCU preceptor training program.

Data from the student surveys were imported into SPSS® for analysis. Comparisons were made between post-training student evaluations of preceptors that had completed training and those that had not completed training. A Mann-Whitney U test was conducted to identify any significant difference between mean student ratings of the precepting skills of trained and untrained preceptors, with statistical significance set at a *p* value of < 0.05. For this purpose, the 18 precepting skills evaluated in the survey were analysed individually and as four grouped categories, based on the four main roles of the preceptor: role model, educator, mentor and assessor [24].

Ethics approval to conduct this study was obtained from the James Cook University Human Ethics Committee (H8276) and informed consent was obtained from all participants of the surveys and interviews.

## Results

### Preceptor post-training survey

**Fig. 1:**
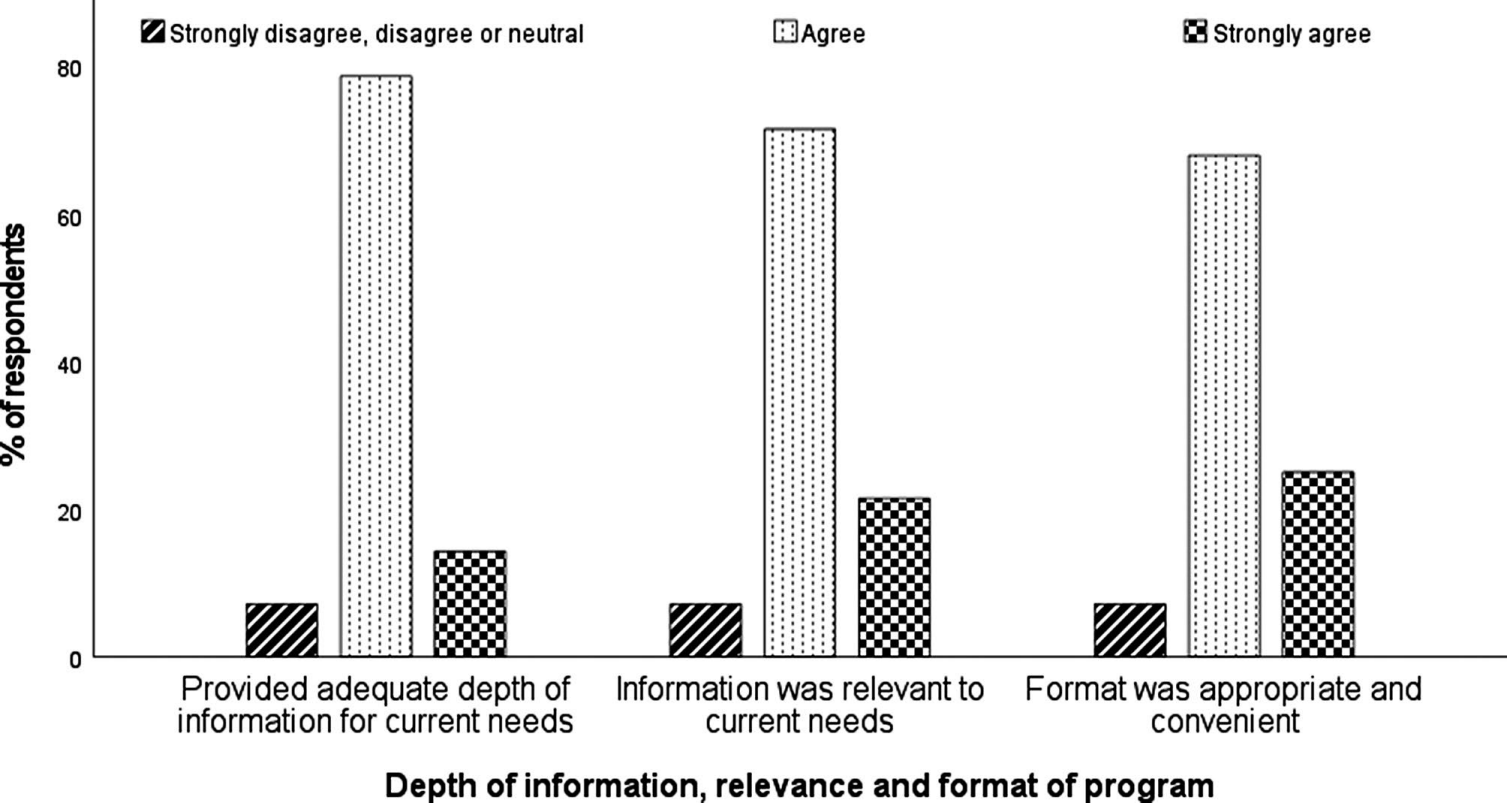
Preceptor ratings on information, relevance and appropriateness of training program (n = 28)

Twenty-eight responses were received for the preceptor survey, representing a response rate of 100%. Overall, the preceptor training program was very well received, with 92.9% of preceptors agreeing or strongly agreeing that the program content had an adequate depth of information and relevance to their current needs and that the format of the program was appropriate and convenient (See Fig. 1).

**Fig. 2:**
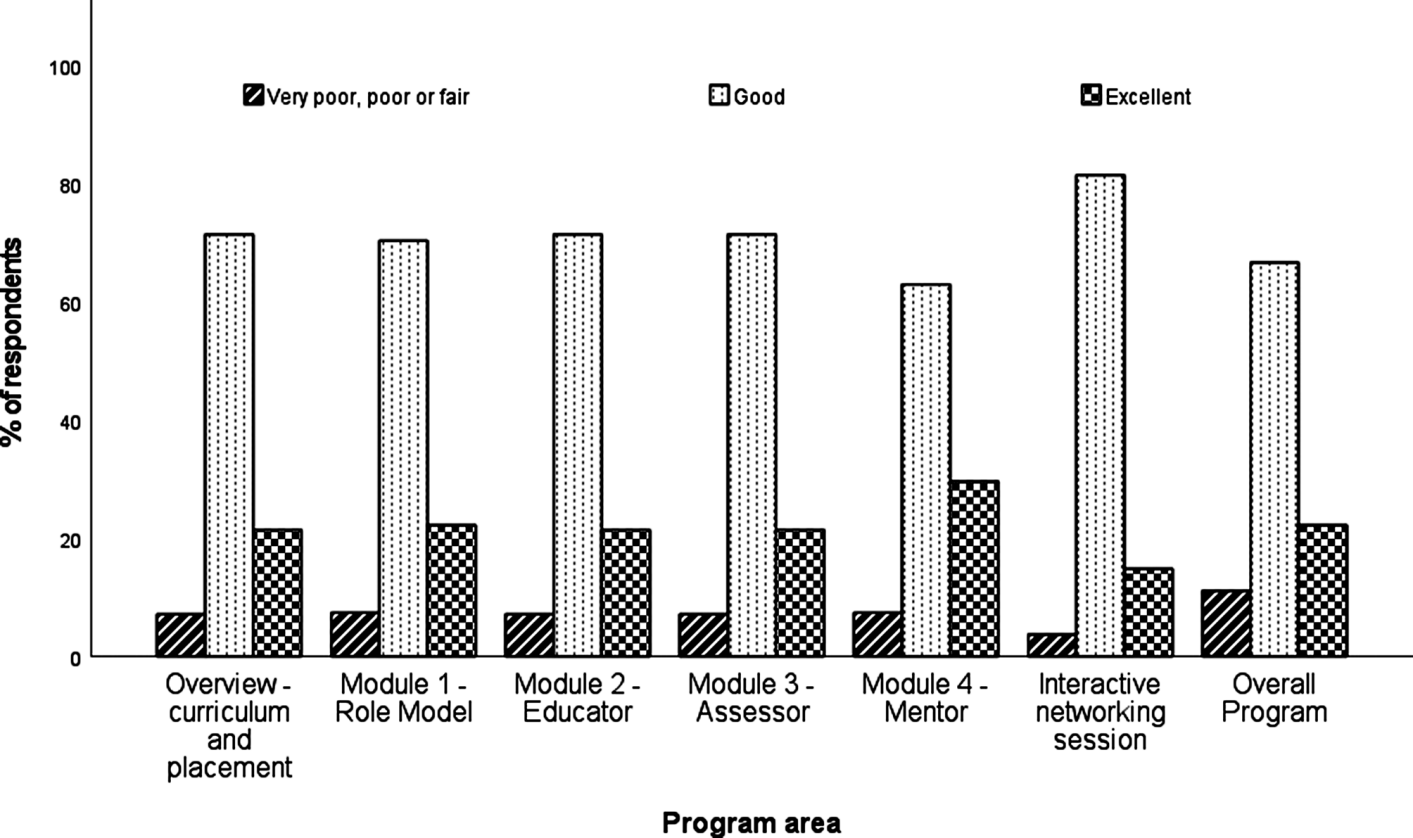
Preceptor ratings of usefulness of areas of the training program (n = 28)

In terms of the individual components of the program, preceptors rated the interactive networking session as the most useful component, with 96.3% of respondents finding this good or excellent. 92.9% of respondents rated Module 2 (Educator) and 3 (Assessor) as good or excellent and 92.6% rated Modules 1 (Role Model) and 4 (Mentor) as good or excellent (See Fig. 2). Regarding the overall program, 22.2% of respondents rated the program as excellent and 66.7% as good, with the remaining 11.1% rating the program as fair and no preceptors rating the program as less than fair.

Chi square analyses did not identify any significant associations between preceptor demographics and their responses to the post-training survey.

### Preceptor post-training survey – content analysis

Content analysis was used to analyse the two open questions in the preceptor survey. Comments on the best aspects of the program were themed into four categories, including program format, program content, networking and training benefits. Seven preceptors reported that the interactive networking session was the best aspect of the program, which correlated with the survey results. Three preceptors commented positively on the program format, appreciating the self-paced nature of the learning and the combination of readings and videos. Three preceptors mentioned specific areas of content which were of particular assistance to them and 3 preceptors spoke about the benefits of the program, with one mentioning that the program had formalized their precepting experience. Table 1 provides a summary of the best aspects of the program and associated quotes.

**Table 1:** Survey content analysis on the best aspects of the training program

Best Aspects	Preceptor quotes
Networking session	*“I thought the networking activity was the most beneficial part of the program in terms of being able to apply the information to specific scenarios but also to gain insights and experiences from other preceptors”.* *“The hour and a half of connecting with other preceptors in the program was great.”* *“Interactive networking”* *“Intersessional active networking”* *“Networking session was great”.* *“The video link with Gillian was great to summarise the program and content”.* *“Talking with other preceptors on how they would handle situations”.*
Format	*“Best taken at own pace and information provided can ensure depth of learning”.* *“It was good that the program was self-paced”.* *“Depth of information”* *“I enjoyed the format of the training - the combination of readings and videos was good”.*
Content	*“I found all of the modules to be of help, strategies given for feedback and the 1-minute preceptor concept helps to leverage the short time instances we have with students in amongst the business of the day where larger chunks of time may not be available - It’s a change of perspective that can help empower preceptor and learner.”* *“How to deal with unmotivated students and problematic behaviour”.* *“Liked positive talk training”.*
Benefits	*“Formalised my experience into a learning platform”.* *“…. and improvements to my scope and practice were highlighted.”* *“The training program is suitable for both experienced and less experienced preceptors”.*

Content analysis on the comments for suggested improvements to the training program can be found in Table 2. Several preceptors commented on the length of time required to complete the program, with two preceptors identifying that some videos used in the modules were not pharmacy specific. Two preceptors noted that course material did not consider the experience of the preceptor, while one preceptor suggested that for preceptors who had not completed their pharmacy degree at JCU, more depth on the JCU curriculum would have been appreciated.

**Table 2:** Survey content analysis on suggested improvements to the program

Suggested improvements	Preceptor quotes
Time consideration	*“Because of the depth, it takes a long time to get through”.* *“Time required to complete”.* *“Consider the time required by practising pharmacists in undertaking this study. The course content was interesting, however, could have been delivered in a much more time efficient manner”.*
More pharmacy specific video examples	*“I found the medical videos very interesting though and they certainly highlighted communication skills but believe this part of the programme could be improved with swapping over to pharmacy specific videos”.* *“Would like to have more examples and videos on training highlighting more pharmacy specific roles”.*
Consider the more experienced preceptor	*“Course material was a little basic in some areas particularly for experienced preceptors”.* *“Program should be considerate of the amount of experience the preceptor already has”.*
More on JCU curriculum for non-JCU graduates	*“A little more depth on the JCU pharmacy curriculum would be good as I am not a JCU graduate”.*

Overall, the course was regarded by preceptors as professional, interesting, and provided a different perspective on precepting.

A lot of information on topics I didn’t think would have that much information to publish. Gave a different perspective on ideas.

Professional and well done.

The course content was interesting.

### Preceptor post-training post-placement interviews

Ten interviews were conducted with preceptors who had completed the training program and subsequently hosted a student. Interviews ranged from 12.5 to 27.5 min, with an average length being 17.5 min. Precepting experience ranged from less than one year to 22 years, with preceptors having hosted between one and 50 students each, as well as from none to more than 20 intern pharmacists. Six of the ten preceptors had received no previous training, with three having completed or partially completed an online intern preceptor training program through the Pharmaceutical Society of Australia or the Pharmacy Guild of Australia, with one completing an online student preceptor training program in 2005 (unsure of which university). All preceptor responses were coded into overall categories and inductive thematic analysis was used to identify themes within these categories. Four categories were coded, including preceptor experiences with their first student post-training, outcome of training on attitudes and practices, effect of training on preceptor confidence levels, and suggestions for future training programs. A summary of the key themes identified in the post-training interviews is provided in Table 3.

**Table 3:** Summary of preceptor post-training interview analysis key themes

**1. Preceptor experiences with their first post-training student**
No major issues
Time management can be a challenge - addressed by careful planning and adequate staffing.
The start of placement can be difficult – planning for the student & establishing a routine.
Difficult to provide challenges for highly performing students.
**2. Effect of training on preceptor attitudes and practices**
Attitudes
Training was beneficial regardless of the experience of the preceptor.
Appreciating the importance of giving the student a better placement experience
Initial orientation and setting expectations are important for both preceptors and students.
Course overview helps to identify student level of knowledge.
More conscious of providing regular feedback throughout the placement.
Practices
Networking allowed for sharing of ideas and learning from each other’s experiences.
Enhanced teaching skills have led to improved lines of communication.
Better insight into the student perspective and ways to manage students.
Improved understanding of feedback techniques – videos were useful.
Better understanding of the student evaluation form and providing honest student appraisal
Involving students in the feedback process
**3. Effect on preceptor confidence levels**
More confident in:
Precepting ability – reinforcement of knowledge for experienced preceptors
Planning for a student and setting expectations
Tailoring precepting to the student
Managing students
Providing constructive feedback
Discussing evaluation reports with students
**4. Suggested areas for future training**
Refresher every few years for experienced preceptors
More on placement requirements and student level of knowledge/skills
Preceptor checklist from the university to ensure that all requirements are completed.
More input into the design of placement activities
Regular communication from the university during placements
Generational differences, student conflict, challenging high performers

#### Preceptor experiences with their first post-training student

All preceptors reported no major challenges with their last placement student, with all progressing smoothly and students being motivated, professional, having a good work ethic and being keen to participate in different roles. Most preceptors agreed that time management could be a challenge at times, particularly during busy periods in the pharmacy. However, this was effectively addressed by careful planning and ensuring that adequate staff were available, allowing them to spend structured time with their student. One preceptor had an issue trying to make up lost time for a student who was ill, whilst another preceptor had to juggle an increased workload due to public holidays and having a locum pharmacist.

I find the main challenge is making sure that the student has a fulfilling experience and seeing as much of the pharmacy as possible while not interfering with any workflow …. Preceptor 6, 3 years of precepting.

Several preceptors identified the start of placement as the most difficult time when the challenge was in adapting to the level of the student, deciding on what to do with the student and establishing a routine. One preceptor commented that their student required some encouragement to communicate with patients. They also were critical of their student completing written placement activities during placement, which they considered to be a less valuable use of placement time, where they should focus on the more clinical aspects of pharmacy practice. Another preceptor who hosted a year 4 student found it difficult to provide challenges and appropriate feedback due to the already high standard of their student.

#### Effect of training on preceptor attitudes and practices

Generally, preceptors found that the training program provided a solid foundation for their precepting, which was beneficial to all preceptors, regardless of experience. Preceptors reported being now alerted to the quality of their precepting and providing a high standard of placement experience for students.

…. It’s made me more keenly aware to make sure that they were having the right type of placement experience. Preceptor 10, 20 years of precepting.

The networking session was positively received, with participants finding significant benefit from sharing their precepting stories and learning from each other’s experiences, particularly those with different backgrounds in terms of both work environment and from a generational perspective. This was thought to promote bonding with other preceptors and further networking in the future. One first-time preceptor commented that as a new preceptor, it was good to see the ‘pitfalls’ of precepting before they happen.

Preceptors were reminded by the program to take time to conduct an initial orientation, which can assist in gaining a better knowledge of the level of the student, setting preceptor expectations and identifying what the student wants to achieve from their placement. The JCU course overview was found to be very helpful in gauging the level of knowledge of the student, with one preceptor commenting on the difficulty in obtaining this information directly from the students. One preceptor also felt that the student level should also include their level of confidence and individual values, which can influence their ability to adapt to the placement environment. Setting expectations was believed to be of importance to ensure a smooth transition into placement and was also helpful in planning student activities. By identifying the knowledge and skills that the student is looking for, preceptors found that they could then tailor their precepting to the needs of the student.

… I felt a lot more comfortable reaching out [to students] to begin with… to ask… ‘This is what I am hoping to achieve, I want to know what you want to achieve … what sort of expectations you have… and this is my expectations.’ That meant there was a lot of transparency between the two of us…. Preceptor 4, 2 years of precepting.

Preceptors reported that the training program had enhanced their teaching ability by improving their lines of communication with their students. The one-minute preceptor technique for teaching and feedback was thought to improve efficiency and lead to better consistency of teaching. (4, 25) Several preceptors mentioned passing this technique on to other pharmacists and even to other staff members. One preceptor believed that while the training program was helpful, their teaching ability had additionally improved with age and experience.

Several comments focused on the impact of the training program on the preceptor’s ability to manage students. It was thought that the program gave them a better insight into the student perspective and their journey. They reported being more aware of the different ways to approach and motivate students, and the generational and other differences that can affect their learning. Training provided a reminder to put aside time for their student, particular mid-placement, and provided additional advice on communication with students to ensure a positive placement experience. Although most preceptors reported no conflict during their last placement, it was noted that training on how to manage difficult or challenging students was useful to know for the future.

Training was found to assist preceptors with practical strategies to have productive feedback conversations and preceptors felt more conscious of their approach to the provision of feedback, ensuring that it was constructive, timely and conducted in collaboration with the student. They were more conscious of providing feedback regularly throughout the placement and felt clearer about the expectations of the university about feedback. They reported that their feedback was being well received by their students, with better responses than before program completion. One preceptor identified that the example videos provided in the training program were a useful tool in assisting them to provide better feedback. About the student evaluation process, preceptors felt that they now had a better understanding of the feedback expected from preceptors on their student evaluation form and that the completion of this evaluation form was now a much easier process. They understood that students in most cases appreciated an honest appraisal of their performance and were now less likely to over-rate their student. Although some students were of a high standard already, preceptors recognized the importance of giving students room to grow and providing some direction for their future learning. They also noted the importance of involving the student themselves in the feedback process.

… it gave me more confidence to say … I guess we always sugarcoat everything and try not to upset them but, at the same time, being able to say it effectively gave me more confidence to give them constructive feedback. Preceptor 2, 15 years of precepting.

… It provided me with an opportunity to have some conscious thought on how I was going to approach feedback. Prior to doing the training … as my experience had been as a student, feedback was quite haphazard …. Preceptor 4, 2 years of precepting.

#### Effect of training on preceptor confidence levels

In terms of confidence levels, it was found that following the training program, preceptors felt more confident in planning to receive a student, as well as in setting placement expectations. For the more experienced preceptors, the program formalized and reinforced their prior knowledge, making them more confident in their ability to provide a good precepting environment. Preceptors reported also that training had given them more confidence in managing students and tailoring their precepting to suit the individual learner. In addition, training had also improved their confidence in providing constructive feedback to students and in discussing their evaluation report.

Honestly, I think it was an invaluable program, for myself at least, I don’t think I would have managed as well if I didn’t have the tools available in my toolkit and it definitely gave me more confidence to continue precepting. Preceptor 7, first time precepting.

#### Suggested areas for future training

Most preceptors interviewed were happy with the training program and felt that all relevant areas were well covered. This included experienced preceptors, who felt that while they did have experience, appreciated the formal training to provide reassurance that they were performing at the right level. In terms of future training, it was suggested that experienced preceptors attend a refresher program every few years or a condensed version of the current program.

There were several comments relating to university expectations, where preceptors reinforced their desire for an understanding of the placement requirements and the current level of the student within the curriculum. A preceptor checklist was suggested as a way of ensuring that preceptors were fulfilling all university requirements for the placement. In terms of the placement itself, one preceptor suggested that preceptors should have more input into the design of placement activities and reiterated their need for regular communication from the university during the placement.

Aside from this, one preceptor suggested more information on generational differences and on dealing with student conflict. Another preceptor required further information on how to challenge and further develop students, who were already of a high standard.

### Student evaluation of the preceptor

Of the 49 student surveys collected post-training, 35 were from community pharmacy placements, with 11 of the 35 preceptors evaluated having completed the training program. Mean values of student ratings for 18 different precepting skills and attributes post-training are provided in Table 4, as well as for the 4 grouped preceptor roles.

**Table 4:** Mean student scores of trained and untrained preceptors for preceptors’ skills and attributes (18 items) and 4 role groups (n = 35)

Preceptor skill or attribute	Trained preceptors	Untrained preceptors
M1 - My preceptor was enthusiastic and engaged with me	4.82	4.36
M2 - My preceptor was sufficiently available for me	4.45	4.09
M3 - My preceptor clarified their expectations of me during my placement	4.55	3.82
M4 - My preceptor was able to manage any conflict during my placement	4.90	4.80
**Mentor – Group mean score (M1 + M2 + M3 + M4)**	**4.68**	**4.27**
RM1 - My preceptor was an effective communicator	5.00	4.41 (*p* = 0.039)
RM2 - My preceptor answered my questions clearly and in a timely manner	4.91	4.50
RM3 - My preceptor demonstrated effective patient counselling and clinical decision making	4.90	4.59
RM4 - My preceptor promoted evidence-based practice and continuing professional development	4.90	4.64
**Role Model – Group mean score (RM1 + RM2 + RM3 + RM4)**	**4.93**	**4.54**
E1 - My preceptor understood my needs as a student	4.81	4.36
E2 - My preceptor understood the educational requirements of JCU Pharmacy	4.82	4.05
E3 - My preceptor was an effective teacher	4.82	4.23
E4 - My preceptor was able to adjust their teaching style to suit my needs	4.82	4.24
E5 - My preceptor provided me with autonomy that was appropriate to my level of experience/competence	4.73	4.55
E6 - My preceptor promoted my critical thinking and problem-solving ability	5.00	4.33
**Educator – Group mean score (E1 + E2 + E3 + E4 + E5 + E6)**	**4.83**	**4.29**
A1 - My preceptor assessed my learning appropriately and fairly	4.82	4.43
A2 - My preceptor provided timely, regular, and constructive feedback to me	4.91	4.27
A3- My preceptor was comfortable in providing any poor feedback to me	4.91	4.48
A4 - My preceptor used online technology effectively when assessing me and providing feedback	5.00	4.60
**Assessor – Group mean score (A1 + A2 + A3 + A4)**	**4.91**	**4.45**

*M = Mentor; RM = Role Model; E = Educator; A = Assessor

It was found that there was a significant difference between trained and untrained preceptors in their skill as an effective communicator (Mann Whitney U: Z = -2.061, *p* = 0.039) with trained preceptors having a significantly higher score. When comparing the mean student rating score for the combined group skills of a Role Model, Educator, Mentor and Assessor, there were no significant differences. Students were invited to provide open feedback on their preceptors in the evaluation survey. Three of the 10 student surveys of trained preceptors included open feedback, and this was all positive.

Very positive placement, there’s nothing I can think of to fault. All preceptors were engaging, answered all questions with enthusiasm and were happy to give me honest feedback.

Of the 25 student surveys of untrained preceptors, 12 open comments were given, of which 5 contained some negative feedback, including lack of communication skills of the preceptor, lack of assistance from staff and variable availability of the preceptor during the placement.

## Discussion

This study aimed to evaluate an online pharmacist preceptor training program for community pharmacy preceptors at JCU, from the perspective of both the preceptor and the student, using a preceptor survey and interviews and a post training student survey. The preceptor survey, conducted immediately post-training, revealed highly positive responses about the impact of the program in terms of the depth of information provided, relevance and convenience, with the interactive networking session being regarded as particularly valuable. Preceptor interviews conducted after hosting a student on placement identified numerous self-reported improvements to precepting attitudes and practices, as well as improved confidence levels in many areas of precepting. Trained preceptors also received overall higher ratings than untrained preceptors, which was statistically significant for the preceptors’ skill as an effective communicator.

The positive preceptor feedback from the training program survey was reflective of the quality of the program and was not unexpected, given that program development was underpinned by a comprehensive JCU preceptor needs analysis [20, 21]. This result also aligned with program evaluations reported in the literature, of which the majority were developed based on an examination of current literature or a needs analysis, with evaluation frequently including a preceptor self-reported survey [14, 16–18]. Cerulli and Briceland in 2004 conducted a training program for community pharmacists, who provided Community Pharmacy Advanced Practice Experiences (CPAPEs). This program, consisting of two live interactive six-hour training sessions, was evaluated, with positive feedback on the relevance and interest of the content and a positive effect on preceptor knowledge of pharmaceutical care [25].

While all areas of the training program were received positively, the most useful area of the program was identified as the interactive networking session, which highlights the importance of communication and interaction between preceptors. This finding is consistent with the literature, with preceptors appreciating opportunities for interaction within preceptor development programs [6, 14, 16, 18]. With this study focusing on community pharmacy preceptors, networking opportunities with other preceptors were considered to be of even greater importance, considering that many of the JCU preceptors are located in rural and remote areas and are often the sole pharmacist in the practice [26]. Participants in a master preceptor train-the-trainer program evaluation identified the importance of networking and collaborating with other preceptors to learn from each other’s precepting experiences [15]. The balance between the provision of written resources and participant interaction is also important. In a study by Smith et al., qualitative feedback on a pharmacy preceptor full-day face-to-face development bootcamp revealed that while feedback was very positive, participants also expressed a desire for more session interactivity [16].

Interviews with program participants following their first post-training placement found that the training program had been beneficial to all preceptors, regardless of experience. Conversely, some preceptors did comment in the post-training survey that the program course material could have considered the level of experience of the preceptor. It was noted that these comments came from preceptors with more than 5 years of precepting experience, who had already completed an intern preceptor training course. Many preceptor training programs have considered and addressed the issue of variation in precepting experience and have ensured that their programs are flexible and tailored to suit the experience of the preceptor. Vos et al. developed a comprehensive range of preceptor development modules in a variety of formats, which allowed preceptors to individualize their learning, and this was well received by preceptors [18]. Pogge et al. reported on a teaching and learning curriculum for preceptors and residents, which provided some streamlining of content and allowed for a number of choices in terms of workshops attendance [14]. Feedback from Smith et al’s evaluation distinguished between new and experienced preceptors, with participant feedback suggesting a full-day session for new preceptors and a half-day session for experienced preceptors [16]. This was also the suggestion from JCU preceptors, that experienced preceptors attend a refresher or condensed program every few years.

Most preceptors found that the training program had impacted their attitude towards precepting by giving them a better understanding of the purpose of placement and becoming more conscious of their responsibilities as a preceptor in providing a high quality placement experience. This result was like other literature which identified positive changes to attitudes and motivation towards precepting [15, 18].

Preceptors found that the program had improved their knowledge and skills in many areas of precepting, including assessing the level of knowledge of the student, how to approach and motivate students and assessing and providing feedback. The one-minute preceptor was identified as a useful technique in providing student education and feedback, which aligned with literature findings [4, 27]. Le et al. found that participants identified strategies such as the one-minute preceptor, the provision of constructive feedback and tailoring of the learning experience, which had impacted positively on their precepting skills [15]. Smith et al. found in their preceptor development bootcamp that there were significant improvements reported by preceptors in their provision of direct instructions as well as verbal and written feedback [16]. In the program evaluation conducted by Vos and Trewet on their preceptor development program, it was found that more than 90% of preceptors who completed the core training activity believed that it enhanced their attitudes, knowledge and skills [18].

This study found that preceptors reported an improvement in their confidence levels following training, in a range of areas, including managing students, providing constructive feedback and discussing student evaluation reports. This is aligned with findings in the literature. Le et al. found that their program improved participants’ confidence in engaging student learners and in clinical teaching [15]. Smith et al. reported preceptor confidence levels trending upwards in all areas following the training [16].

Preceptors in this study expressed a need for some direction from the university regarding expectations of preceptors and an overview of the curriculum. The provision of an orientation to precepting is a mandatory requirement of the Accreditation Council for Pharmacy Education (ACPE) [28], however, in O’Sullivan’s survey of current preceptor orientation and development programs in the United States in 2020, it was found that only 65% of Schools of Pharmacy had met the ACPE requirements for preceptor orientation [10]. Preceptors in the JCU evaluation also stated that the program had been a reminder to them that, in addition to preceptor orientation, a student orientation to the placement is important, to identify student needs, set placement expectations and plan student activities.

There are few evaluations of preceptor training programs that include the student perspective [11, 18]. A comprehensive preceptor training program developed in the US was evaluated by program participants (preceptors) as well as by students, using a standardized student evaluation of preceptors, administered both before and after program participation [18]. This study revealed that following training, students rated more preceptors as ‘good’ and less preceptors as ‘fair’ or ‘poor’, while the number of preceptors rated as ‘excellent’ did not change. A 2015 study evaluated three objective structured teaching exercises (OSTEs) from the perspective of both preceptors and a selection of standardized students. This study found a significant increase in preceptor confidence in performing OSTEs, however, the training process was both time and resource intensive. Students agreed with preceptors on areas, where preceptors were least and most confident in providing feedback [11].

From the analysis of the JCU post-training student evaluations, it was found that from the perspective of the student, preceptor skills as an effective communicator were significantly improved by training. The ability to communicate effectively is one of the most important skills for any health professional, but particularly for preceptors, who are not only required to communicate with patients and other healthcare providers, but additionally to establish a professional relationship with the student. It is known that effective communication is not necessarily an innate skill and can improve with training [29]. Good communication skills are considered to be an essential foundation for effective student management, the provision of appropriate feedback and for conflict resolution [30]. The improvement in preceptor communication skills following training, which was identified from the student evaluation data, is consistent with the preceptor post-training interviews, where preceptors reported that the provision of additional tools and techniques to teach, manage and provide feedback more effectively has resulted in improved lines of communication with students.

While not significant, it was observed that student ratings of the preceptor in their ability to promote critical thinking and problem solving (E6) was improved. Critical thinking is the process that facilitates clinical reasoning which, in the context of a healthcare professional, is a skill that enables them to use their existing knowledge to analyse and find solutions to clinical situations, thus putting their knowledge into practice [31]. Pharmacists use this key skill routinely every day when making clinical decisions and solving problems in the practice. Clinical reasoning can be a difficult skill to both describe and teach, however, it is recognized that while much can be learned from observing experienced healthcare professionals in the practice setting, this skill can be further developed by the preceptor through appropriate questioning and discussions with students [32, 33]. This result from the student evaluation survey indicates that the training program may have influenced preceptors by providing teaching strategies, such as the One-Minute Preceptor [32], that promote critical thinking.

One advantage of the study methodology was the use of Kirkpatrick’s model of training evaluation criteria. By including both preceptors and student in the evaluation, this study was able to provide results on not only the impact of the training program on preceptor learning (Kirkpatrick’s levels 1 and 2), but also on the program outcomes, by identifying changes to preceptor behaviours and to student-rated preceptor performance. (Kirkpatrick’s levels 3 and 4)

The major limitation of this study was the low participant numbers. This may have affected the ability of the data to detect significant associations between preceptor demographics and survey responses. Furthermore, as this study involved community pharmacy preceptors only, there are limitations to the generalizability of the findings. Although the preceptor survey was anonymous, there may have been some positive response bias due to the involvement of the principal researcher in conducting the interactive networking sessions, while also being previously known to several of the participants through their association with the university.

While the student survey of the preceptor was also anonymous to promote participation, low overall student numbers at JCU, which were partially attributed to the Covid-19 pandemic, resulted in a lower than expected number of student surveys received for analysis. Furthermore, with placement experiences being hosted at different times throughout the year following preceptor training, the time difference between program completion by the preceptor and the completion of the student survey may have varied. This may have affected the outcome of the student survey, with preceptor performance potentially changing over time.

## Conclusion

This study describing the evaluation of the JCU community pharmacist preceptor training program, has produced positive results. The training program was rated highly by participants, with the opportunity for preceptor interaction being the most favourable feature of the program. Positive changes to self-reported preceptor attitudes, behaviour and confidence levels were identified following this program, and student evaluations reported improved preceptor performance as an effective communicator. There are a limited number of evaluations of pharmacist preceptor training programs available in the literature, with few being evaluated from the perspective of both the preceptor and the student. Further studies which include the student perspective on preceptor performance following training are recommended, to complement post-training preceptor evaluations and ensure a balanced perspective. Through the appropriate design of preceptor training program evaluations, programs will continue to be modified and improved, ensuring a high quality, engaging and professionally relevant training experience for preceptors. This will result in enhanced placement experiences for students and an anticipated improvement in the quality of pharmacy graduates.

## Data Availability

The datasets used during this study are available from the corresponding author on reasonable request.

## Abbreviations

ACPE: Accreditation Council for Pharmacy Education
APC: Australian Pharmacy Council
CPAPE: Community Pharmacy Advanced Practice Experience
JCU: James Cook University
OSTE: Objective Structured Teaching Exercise
US: United States
